# Identification and Validation of Novel Biomarkers for Diagnosis and Prognosis of Hepatocellular Carcinoma

**DOI:** 10.3389/fonc.2020.541479

**Published:** 2020-09-25

**Authors:** Xiaoyi Hu, Mingyang Bao, Jiacheng Huang, Lin Zhou, Shusen Zheng

**Affiliations:** ^1^Division of Hepatobiliary and Pancreatic Surgery, Department of Surgery, The First Affiliated Hospital, Zhejiang University School of Medicine, Hangzhou, China; ^2^National Health and Family Planning Commission of China Key Laboratory of Combined Multi-Organ Transplantation, The First Affiliated Hospital, Zhejiang University School of Medicine, Hangzhou, China; ^3^Key Laboratory of the Diagnosis and Treatment of Organ Transplantation, Research Unit of Collaborative Diagnosis and Treatment for Hepatobiliary and Pancreatic Cancer, Chinese Academy of Medical Sciences, Hangzhou, China; ^4^Key Laboratory of Organ Transplantation, The First Affiliated Hospital, Zhejiang University School of Medicine, Hangzhou, China; ^5^State Key Laboratory of Genetic Engineering, School of Life Sciences, Institute of Biostatistics, Fudan University, Shanghai, China

**Keywords:** prognosis, biomarker, GEO, hepatocellular carcinoma (HCC), dignosis

## Abstract

**Introduction:** Hepatocellular carcinoma (HCC) is one of the leading causes of cancer-related deaths worldwide due to poor survival outcome. Thus, there is an urgent need to identify effective biomarkers for early diagnosis and prognosis prediction.

**Methods:** A total of 389 differentially expressed genes (DEGs) between HCC samples and normal were selected based on the Robust Rank Aggregation (RRA) method. We combined DEGs expression and clinical traits to construct a gene co-expression network through WGCNA. Forty hub genes were selected from the key module. Among them, YWHAB, PPAT, NOL10 were eventually identified as prognostic biomarkers using multivariate Cox regression model. Biomarkers expression pattern was investigated by informatic analysis and verified by RNA-seq of 32 patients with HCC. DiseaseMeth 2.0, MEXPRESS, and Tumor Immune Estimation Resource (TIMER) were used to assess the methylation and immune status of biomarkers. GSVA, CCK8, colony formation assay, Edu imaging kit, wound-healing assay, and xenograft tumor model were utilized to investigate the effects of biomarkers on proliferation, metastasis of HCC cells *in vitro*, and *in vivo*. The Kaplan–Meier (KM) plotter and ROC curves were used to validate the prognostic and diagnostic value of biomarker expression.

**Results:** All the selected biomarkers were upregulated in HCC samples and higher expression levels were associated with advanced tumor stages and T grades. The regulation of YWHAB, PPAT, NOL10 promoter methylation varied in tumors, and precancerous normal tissues. Immune infiltration analysis suggested that the abnormal regulations of these biomarkers were likely attributed to B cells and dendritic cells. GSVA for these biomarkers showed their great contributions to proliferation of HCC. Specific inhibition of their expression had strong effects on tumorigenesis *in vitro* and *in vivo*. ROC and KM curves confirmed their usefulness of diagnosis and prognosis of HCC.

**Conclusions:** These findings identified YWHAB, PPAT, and NOL10 as novel biomarkers and validated their diagnostic and prognostic value for HCC.

## Introduction

Primary liver cancer is the fourth leading cause of cancer-related deaths worldwide and hepatocellular carcinoma (HCC) accounts for the majority of cases (1, 2). At present, it is difficult to accurately predict prognosis in patients with HCC because of cirrhosis and additional comorbidities (3). Tumor burden and stage of liver dysfunction proved to be important indictors in prognosis prediction and treatment selection (4, 5). Multiple cohort studies and randomized controlled trials have shown the survival benefits from several therapeutic strategies including liver resection (LR), tumor ablation (ABL), liver transplantation (LT), intra-arterial therapy (IAT), and immune checkpoint inhibitors therapy (4–6). However, most patients are not diagnosed until the tumor progressed to the late stage, missing the best period for treatment (7, 8). The prognosis of liver cancer remains poor overall, with a 5-year survival of 18% (9). Therefore, it is necessary to search for effective diagnostic and prognostic biomarkers for HCC.

Cancer biomarkers are defined as diagnostic indicators for tumor biological behavior and stage assessment. To date, more than 25 molecular therapies in 526 oncology have been approved for clinical use based on predictive biomarkers (10). In HCC, the landscape of molecular characterizations has uncovered targetable drivers, of which ~25% are considered to have potential functions (11, 12). Recent clinical evidence indicates that the percentage of patients in HCC with genomic alterations related to response is approaching 20% (13). The identification of recurrently mutated genes and copy number alterations have shown that mutations and prevalent drivers (TERT, CTNNB1, TP53, AXIN1, ARID1A, and ARID1B) detected in HCCs can be considered as candidates for biomarkers. However, the advancements in the understanding of these molecular drivers have not yet been translated into biomarker-driven trials of precision medicine. Further studies are required to identify novel specific biomarkers of HCC to improve the prognosis. Fortunately, rapid development of genome sequencing and bioinformatics provides good opportunities for researchers to integrate information from multiple independent studies. Large sample sizes overcome substantial inter-study heterogeneity and enhance credibility in promising biomarkers exploration.

The aim of this study was to explore potential diagnostic and prognostic biomarkers and their biological functions in the progression of HCC. In our study, 10 eligible Gene Expression Omnibus (GEO) datasets were integrated with Robust Rank Aggregation (RRA) method to identify robust differentially expressed genes (DEGs) between HCC and adjacent normal tissues. We combined expression profile of these DEGs and clinical characteristics of the samples from The Cancer Genome Atlas (TCGA) database to find hub genes through weighted gene co-expression network analysis (WGCNA). Multivariate Cox regression with forward stepwise strategy was used to select biomarkers with prognostic value from hub genes. In addition, association of biomarker expression with methylation status and immune infiltration were detected. The result of Gene Set Enrichment Analysis (GSEA) revealed that biomarkers were closely associated with tumor proliferation, which was further validated through their functional roles in the progression of HCC both *in vivo* and *in vitro*. Kaplan–Meier (KM) survival curves and receiver operator characteristic (ROC) curves were drawn to confirm prognostic and diagnostic value of biomarkers at last. The bioinformatic analysis of HCC in this study provides insight into the critical roles of biomarkers in cell proliferation, diagnosis, and prognosis of HCC.

## Materials and Methods

### Selection Strategy of GEO Datasets

We searched for datasets related to HCC from Gene Expression Omnibus (GEO) (http://www.ncbi.nlm.nih.gov/geo/) with the Mesh terms “liver neoplasms” and “human.” A further filter was performed with organism “Homo sapiens” and study type “Expression profiling by array.” Consequently, 730 datasets were included. In order to guarantee the quality of this study, datasets obtained above were excluded if the following terms were met:

1. Sample counts lower than 10
2. Non-tumor liver specimens obtained from patients with chronic hepatitis or cirrhosis.

According to the systematic screening strategy, nine series accessions were finally included in this study: GSE14520, GSE22405, GSE62232, GSE59261, GSE101685, GSE121248, GSE64041, GSE50579, and GSE25097 (Table 1). In the GSE14520, part of samples was carried out on the Affymetrix Human Genome U133A 2.0 Array (GPL571) while other samples were processed on the 96 HT HG-U133A microarray platform (GPL3921). Therefore, GSE14520 was divided into two datasets, namely GSE14520571 and GSE145203921. In addition, transcriptome profiling and clinical information of primary tumor and solid normal tissue were downloaded from the TCGA-LIHC database (https://cancergenome.nih.gov/).

**Table 1:** Characteristics of GEO datasets included in the study.

**Series accession ID**	**Country (region)**	**Number of samples**		**Platform ID**
		**Tumor**	**Normal**	
GSE101685	Taiwan	24	8	GPL 570
GSE121248	Singapore	70	37	GPL 570
GSE59261	Italy	8	8	GPL18541
GSE62232	France	81	10	GPL570
GSE25097	United States	268	243	GPL10687
GSE50579	Germany	67	10	GPL14550
GSE64041	Switzerland	60	60	GPL6244
GSE14520	United States	22	19	GPL571
GSE14520	United States	225	220	GPL3921
GSE22405	United States	24	24	GPL10553

*GSE, Gene Expression Omnibus Series; GPL, Gene Expression Omnibus Platform*.

### Screening for DEGs

We downloaded the series matrix files of datasets from GEO. The R package “limma” was utilized to normalize the data, average character values of duplicate genes and remove duplicate (14). We then used ‘RobustRankAggreg’ R package to integrate the results of those 10 datasets to find the most robust DEGs (15). The log_2_ FC of each gene indicated its ranking in the final gene list, and genes with adjusted *P* < 0.05 and |log_2_ FC| ≥ 1 were considered as significant DEGs in the RRA analysis.

### GO and KEGG Enrichment Analysis

We conducted Gene Ontology (GO) enrichment and Kyoto Encyclopedia of Genes and Genomes (KEGG) pathway analyses using the R package “clusterprofiler” (16). Bubble charts visualized the top 10 terms of the biological process, cellular component and molecular function of DEGs, respectively. A visual network map of signaling KEGG pathway was further drawn in Cytoscape 3.7.1 software (17).

### Identification of Hub Genes

We extracted the top 5144 DEGs from RRA analysis to perform WGCNA with clinical and expression data from TCGA. The “WGCNA” R package was applied to construct clinical traits-related modules and identify hub genes (18). The adjacency matrix was transformed into topological overlap matrix (TOM). Genes were divided into multiple gene modules based on the TOM–based dissimilarity. Here, soft-thresholding power was set as 7 (scale free R^2^ = 0.85), cut height as 0.25, and minimal module size as 30. Correlations between module eigengenes and clinical traits were calculated. Moreover, gene significance (GS) was defined as the absolute value of the correlation coefficient between genes and traits, and module membership (MM) of the gene in the module was deemed to be the relation to expression profiles. The gene with GS >0.2 and MM >0.8 in the module most relevant to clinical traits was considered hub one. The function annotation of hub genes was investigated through GO enrichment analysis.

### Construction of Prognostic Biomarkers

Based on the optimal cutoffs estimated by X-tile 3,6,1 (Yale University, New Haven, CT, USA), continuous variables, expression profiles of hub genes, were transformed into dichotomous variables, low- and high-expression group. These categorical variables along with clinicopathological factors, such as tumor stage, T grade, age, body mass index (BMI), and gender, were included to establish a multivariate Cox regression model in the TCGA-LIHC dataset. Moreover, forward stepwise strategy was used to select most robust independent prognostic variables. Based on the above results, we built a nomogram to visualize the prognosis prediction process and evaluated the prediction accuracy through the calibration curves.

### RNA Extraction, RNA-seq, and Data Analysis

RNA was extracted from liver fresh frozen tissues of 32 patients treated in our hospital, The First Affiliated Hospital of Medical School of Zhejiang University (FAHMSZU). DNA libraries were sequenced by Illumina HiSeq 2500 (pair-end 150-nucleotide read length). Raw data were deposited in NCBI Gene Expression Omnibus (GSE138485/PRJNA576155). HISAT2 (version 2.1.0) was used to align Sequencing reads human reference sequence (UCSC/hg38.p12) (19). FeatureCounts (release 1.6.3) was performed for ach gene count from trimmed reads against the GENCODE (release 30) (https://www.gencodegenes.org) transcript models (20).

### Correlations Between Biomarker Expression With Clinical Features, Methylation, and Immune Infiltration

To further evaluate the prognostic value of selected biomarkers, violin plots were depicted to compare the expression levels of biomarkers in tumor tissue and normal in the TCGA dataset and the FAHMSZU cohort. Moreover, tumor samples were divided into several subgroups based on pathological stage and T grade to assess correlations between expression levels and clinical characteristics. In addition, we investigated differences in methylation levels of biomarkers with between normal and tumor samples using UALCAN (https://ualcan.path.uab.edu/index.html) online tool (21), which is a comprehensive web resource for analyzing epigenetic regulation of gene expression by promoter methylation in the TCGA database. Furthermore, MEXPRESS (http://mexpress.be) (22), an online tool for visualizing expression, DNA methylation and clinical TCGA data, was used to explore association between biomarker expression and methylation levels at multiple DNA sites. In terms of the relationship between biomarker expression and tumor-infiltrating immune cells, the online tool TIMER (https://cistrome.shinyapps.io/timer/) was utilized (23), which is a comprehensive resource for the clinical relevance of tumor-immune infiltrations.

### Gene Set Enrichment Analysis (GSEA)

GSEA of biomarkers was performed to find potential functions based on the “clusterprofiler” R package (16). The gene set “c2.cp.kegg.v6.2.symbols.gmt,” downloaded from the Molecular Signature Database (MSigDB, http://software.broadinstitute.org/gsea/msigdb/index.jsp), was selected as the reference gene set. Tumor samples were classified into two groups as described in section Construction of Prognostic Biomarkers.

### Validation of Diagnostic and Prognostic Value of Biomarkers

Kaplan–Meier (KM) survival curves were drawn between high- and low-expression biomarker groups to show prognostic significances of biomarker expression in patients with HCC based on the KM plotter database (24). In addition, diagnostic value of prognostic biomarkers was assessed by receiver operating characteristic (ROC) curves and the area under the curve (AUC) in the TCGA dataset and the FAHMSZU cohort.

### Cell Culture and Transfection

Four HCC cell lines (LM3, Huh7, 97H, SK-hep1) and one normal hepatocyte cell line (LO2) were purchased from the Liver Cancer Institute of Fudan University (China). All HCC cells lines were cultured in DMEM high glucose medium (Biological Industries, IL with 10% FBS and LO2 were cultured in RMPI 1640 medium (Biological Industries, Israel) with 10% FBS at 37°C in a humidified incubator containing 5% CO2. LM3 and Huh7 cells were transfected with particular siRNAs purchased from GenePharma (China) using lipofectamine2000 (Invitrogen, USA). shRNA-YWHAB was constructed according to the sequence of YWHAB si-1#. LM3 cells were infected by lentivirus at a MOI of 20 pfu per cell. The stable transformants were selected with 4 μg/ml puromycin.

YWHAB si-1#: Sense: GCUGAAUUGGAUACGCUGAAUTT
Antisense: AUUCAGCGUAUCCAAUUCAGCTT
YWHAB si-2#: Sense: CGCUGAAUGAAGAGUCUUAUATT
Antisense: AUAUAAGACUCUUCAUUCAGCGTT
PPAT si-1#: Sense: CCCUUCGUUGUUGAAACACUUTT
Antisense: AAGUGUUUCAACAACGAAGGGTT
PPAT si-2#: Sense: GUAGCUUCACCACCAAUUAAATT
Antisense: AUUUAAUUGGUGGUGAAGCUACTT
NOL10 si-1#: Sense: GCUGCGGAGAAUAAUGUUUTT
Antisense: AAACAUUAUUCUCCGCAGCAT
NOL10 si-2#: Sense: GCACAGUCUAUGAUGAUUATT
Antisense: UAAUCAUCAUAGACUGUGCTT.

### RNA Extraction and qRT-PCR Analysis

RNA was extracted from the cell lines using TRIzol reagent (Invitrogen, USA). RNA s μg) was then reversely transcribed into cDNA through a Reverse Transcription Kit (Takara, China). SYBR Green (Takara, China) was used for real-time PCR analysis. The results were normalized to the expression of glyceraldehyde-3-phosphate dehydrogenase (GAPDH).

#### Western-Blot

Scholars extracted the total proteins for 60 min on ice in RIPA buffer (Thermo Scientific, USA) having protease and phosphatase as an inhibitor (Cell Signaling Technology, USA). Cell lysates were centrifuged at 1.2 × 10^4^ g, 4°C for 15 min, and the concentrations of supernatants were examined with a BCA Protein assay kit (Thermo Scientific, USA). 30 μg protein was separated by 10% SDS-PAGE (Life Technology, USA) and then moved to 0.45 μm PVDF membranes (Millipore, USA). After that, the PVDF membranes were instantaneously obstructed by 5% defatted milk in 1 × tris-buffered saline (pH 7.6, 50 mM Tris and 150 mM NaCl) for about 2 h at a regular temperature. The membranes were incubated with monoclonal antibodies at 4°C for 24 h. In total, primary antibodies included those for YWHAB (Abcam, UK, ab32560), NOL10 (Abcam, UK, ab181161), PPAT (Abcam, UK, ab125864), GAPDH (Abcam, UK, ab8245). We washed membranes for three times then incubated with fluorescent-tagged secondary antibody for another 2 h. Then, we used electrochemiluminescence (ECL) to read and the immunoblots were examined with a visible imaging system (Bio-Rad, USA).

### Colony Formation Assay and Cell Viability

A total of 2 × 10^3^ siRNA-transfected cells/well were plated into 6-well plates at 37°C in a humidified incubator containing 5% CO2. After 14 days, the cells were stained and photographed. Cell proliferation was detected by using a Cell Counting Kit-8 assay (Dojindo, JPN). Transfected LM3 and Huh7 cells (2 × 10^3^/well) were plated into 96-well plates. We measured the absorbance at 450 nm. The ethynyl deoxyuridine assays used an EdU Apollo 567 imaging kit (RiboBio, CN) to determine cell viability.

### Cell Migration, Invasion, and Wound-Healing Assay

Transfected Huh7 cell (3 × 10^4^/well) in media with 1% FBS were added to the upper insertion chamber (Millipore, USA), while the lower chamber contained the medium with 10% FBS. After incubation for 24 h, the remaining cells on the upper membrane were removed with a cotton swab. Cells migrating through the membrane were dyed with methanol, stained with 0.1% crystal violet. Cell invasion assay was performed using a 24-well Transwell chamber. At 24 h after transfection, transfected Huh7 cell were transferred to the Matrigel-coated top chamber containing 100 mL of serum-free medium. FBS was added to the bottom chamber as chemoattractant. After 24 h, cells on the bottom of the chambers were fixed in 4% methanol and stained with 0.1% crystal violet. At 24 h after transfection, transfected Huh7 and LM3 cell was created in the center of each well using a micropipette tip. Cell migration was assessed by measuring the movement of the cells into the scratch in the well. The wound-closure speed after 24 and 48 h was determined and normalized to the length at 0 h.

### Tumor Xenograft Model

HCC LM3 cells were harvested after being transfected with shRNA or an empty vector. Next, the cells (4 × 10^7^) were injected subcutaneously into the immunodeficient mice (Shanghai XeB Animal Ltd, CN) (six animals per group). Eighteen days later, all mice were sacrificed, and tumor tissue was collected for analysis. Tumor volume was calculated using the formula: V (mm^3^) = Width^2^ (mm^2^) × length (mm)/2. The Zhejiang Medical Experimental Animal Care Commission approved the experimental protocols involving animals.

### Statistical Analysis

All statistical analyses were performed in R 3.6.1 software and web resources. The non-parametric Wilcoxon rank-sum test was applied to compare mRNA expression in tumor and normal tissues and Kruskal–Wallis test was used to compare expression differences between multiple tumor stage or T grades samples and normal samples. A multivariable regression model with biomarker expression and infiltration of immune cells was constructed to calculate partial correlation coefficients between independent variables in TIMER tool. Fisher's exact test was adopted to determine gene enriched pathway and process in DAVID functional annotation tool. Log-rank test was used to show survival differences in the KM curves. All assays were carried out three times. The Student's *t*-test was used to examine the statistical significance of differences between two groups if the data were normally distributed; otherwise, the Mann-Whitney test was used. A 2-sided α of <0.05 was considered statistically significant.

## Results

### Identification of DEGs

**Figure 1:**
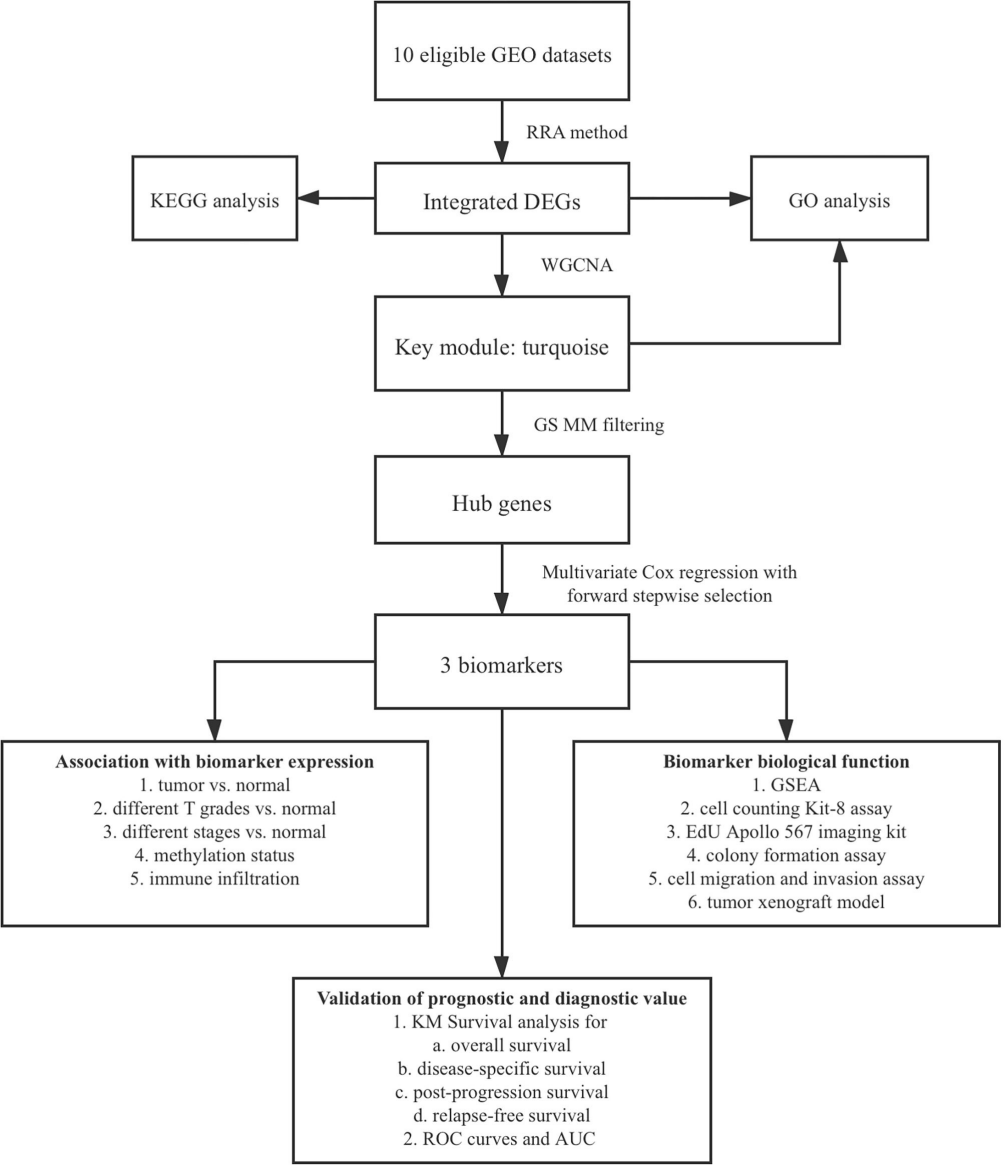
Flow chart of biomarker identification and validation.

**Figure 2:**
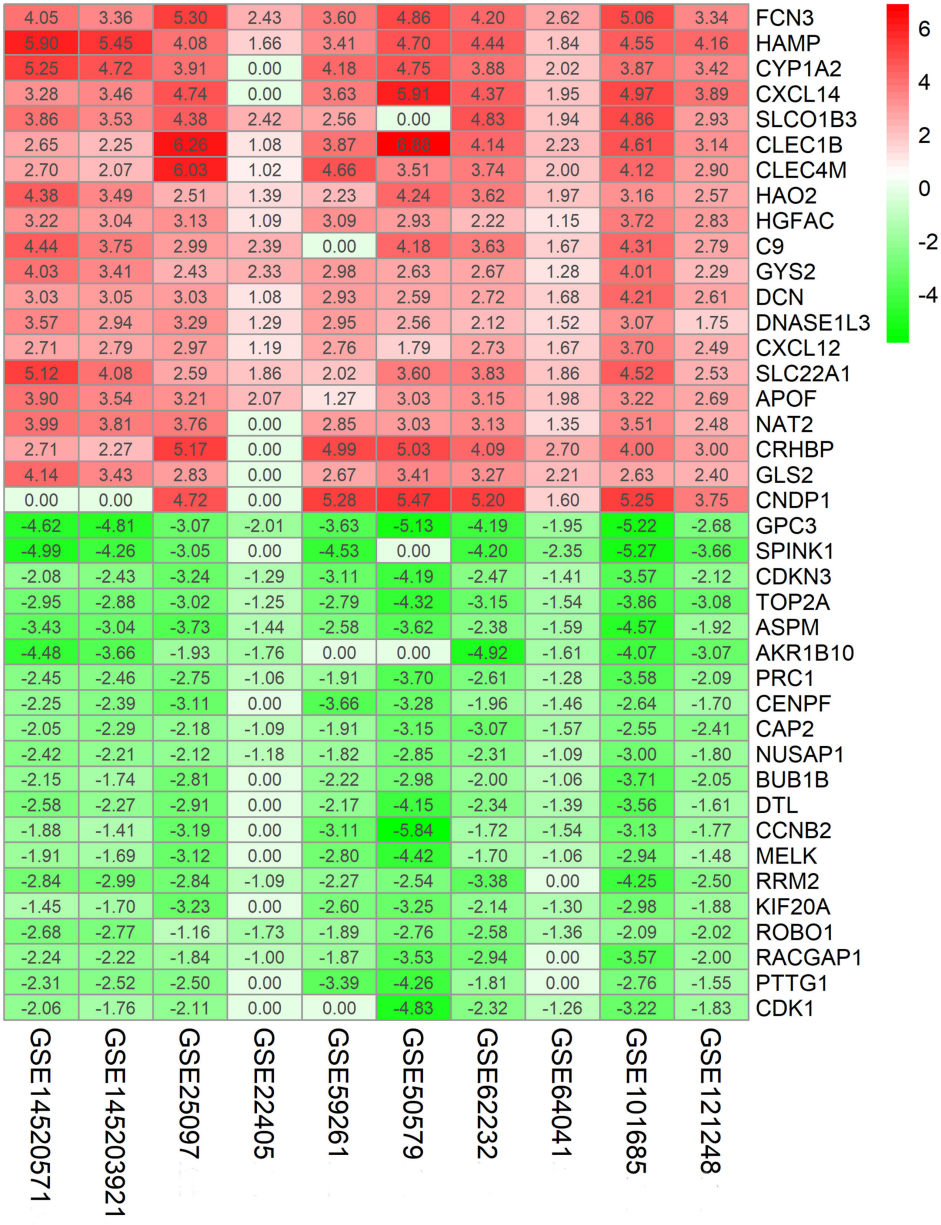
Identification of robust DEGs by RRA method. Heatmap shows the top 20 upregulated and downregulated DEGs in GEO series accessions. Each row denotes one DEG and each column represents one dataset. The color changes from red to green indicates regulation from up to down. The numbers in the box stand for logarithmic fold change.

The flowchart shows identification and validation process of biomarkers with diagnostic and prognostic value (Figure 1). As a result, ten eligible datasets that met screening criteria were included in the subsequent analysis. Detail information of included datasets, such as GEO series accession ID, study country, sample information, platform ID, was shown in Table 1. All of ten expression matrices were sorted by logarithmic fold change (log_2_ FC) ranking of internal genes and integrated to screen robust DEGs. Finally, 389 integrated DEGs, including 292 upregulated and 97 downregulated significant DEGs, were identified based on the RRA analysis. The top 20 upregulated and downregulated DEGs were drawn on the heatmap (Figure 2). CLEC1B was the most significant upregulated gene (log_2_ FC = 6.88) while CCNB2 ranked first among all of downregulated genes (log_2_ FC = 5.84).

### Functional Enrichment Analysis of DEGs

**Figure 3:**
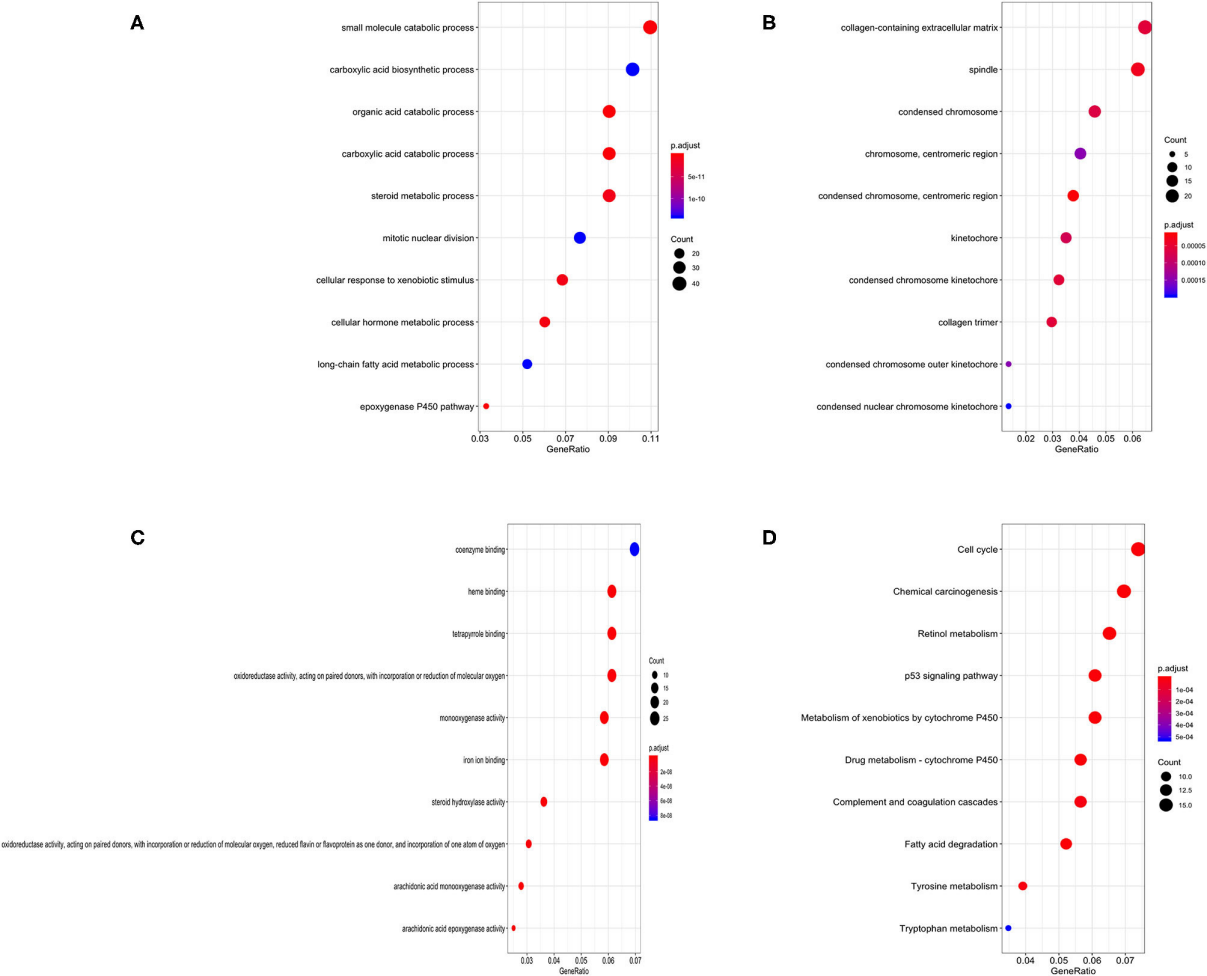
Bubble charts of enriched GO annotations and KEGG pathways for integrated DEGs. **(A)** The top 10 enriched biological process GO terms. **(B)** The top 10 enriched cellular component GO terms. **(C)** The top 10 enriched molecular function GO terms. **(D)** The top 10 enriched KEGG pathways.

Gene Ontology (GO) annotation was performed for the significant integrated DEGs. The top biological process GO enrichment terms were small molecule catabolic process, carboxylic acid biosynthetic process, and organic acid catabolic process. Collagen-containing extracellular matrix was the most enriched cellular component GO terms, followed by spindle and condensed chromosome. In addition, we found several enriched molecular functions such as coenzyme binding, heme binding, and tetrapyrrole binding. What's more, the Kyoto Encyclopedia of Genes and Genomes (KEGG) pathways in which integrated DEGs were most enriched were cell cycle, followed by chemical carcinogenesis, retinol metabolism, p53 signaling pathway, and metabolism of xenobiotics by cytochrome P450 (Figure 3). The result of KEGG pathway enrichment was further visualized on network diagram drawn by Cytoscape software, as shown in Supplementary Figure 1.

### WGCNA and Module Analysis

**Figure 4:**
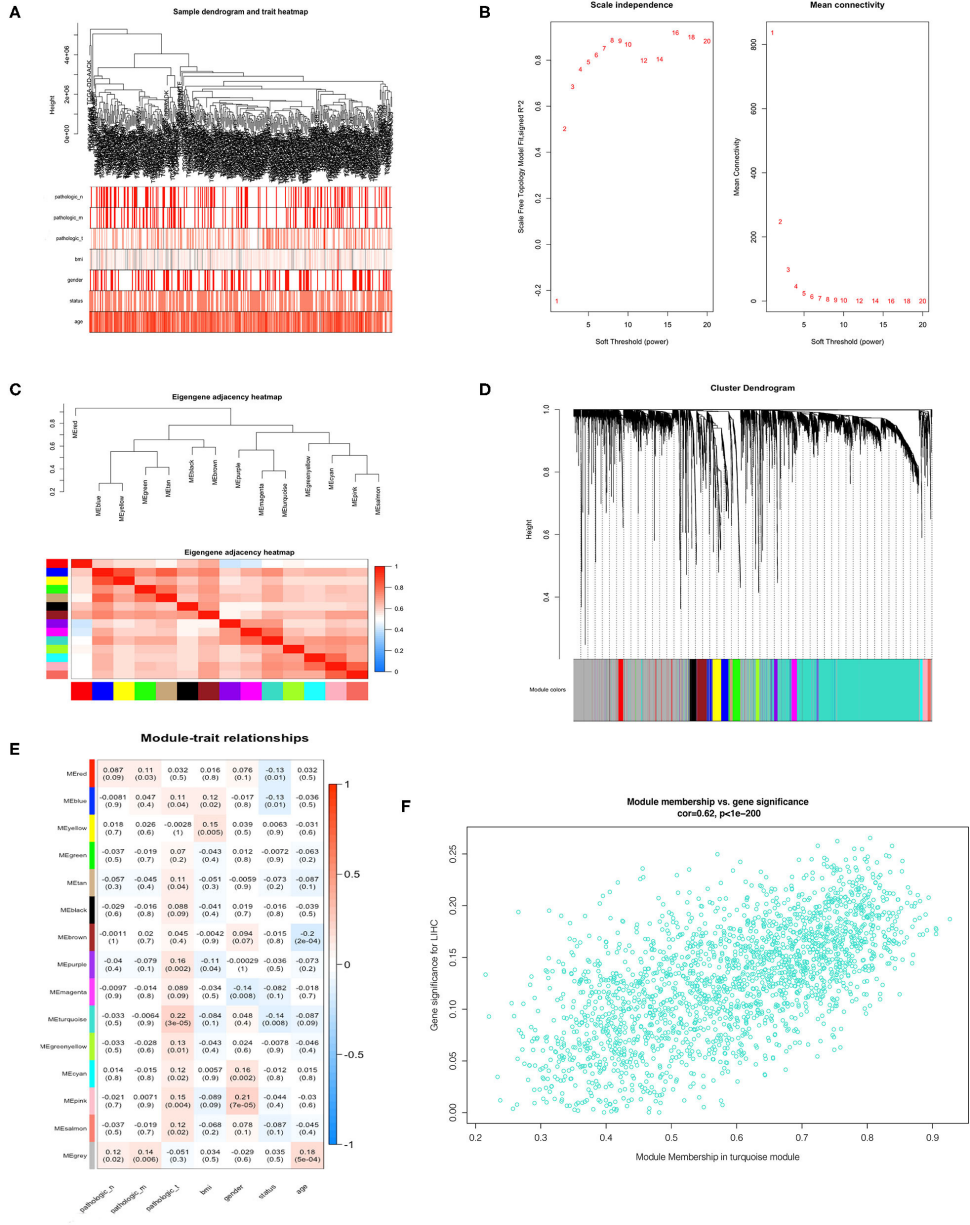
Identification of hub genes from key modules associated with clinical traits in the TCGA-LIHC dataset through WGCNA. **(A)** Clustering dendrograms of robust DEGs from RRA analysis and clinical traits of TNM grade, BMI, gender, and age displayed at the bottom. **(B)** Analysis of the scale-free fit index and the mean connectivity for various soft-thresholding powers. **(C)** Clustering of module eigengenes. **(D)** Dendrogram of all DEGs clustered with dissimilarity measure based on topological overlap. **(E)** Heatmap of the correlation between module eigengenes and clinical traits. Each row denotes a module eigengene, each column represents a clinical trait and each cell contains the correlation coefficient and *P*-value. **(F)** Scatter plot of genes in turquoise module.

We incorporated the expression profile of integrated DEGs with clinical traits of the TCGA-LIHC samples to construct a gene co-expression network. Clinical characteristics including TNM grade, BMI, age, gender, and living status of HCC samples were clustered with expression matrix as shown on the heat map (Figure 4A). Next, the soft threshold was set to 7 so that the scale-free network evaluation coefficient R2 reached 0.85 for the first time (Figure 4B). A total of 15 modules were identified from the co-expression network after merging similar modules according to a cut height of 0.25 (Figures 4C,D). Correlations between module eigengene and clinical traits were visualized in Figure 4E. We found the turquoise module most relevant to clinical traits from the heatmap of module-trait relationships, especially the T grade (correlation coefficient = 0.22, *P* = 3E−06) (Figure 4F). Forty hub genes were identified from the turquoise module by setting the screening criteria of GS >0.2 and MM >0.8. Hub genes showed a close connection with each other, as shown in Supplementary Figure 2A. What's more, GO function analysis was performed to reveal potential biological functions of hub genes. As a result, viral process was the most significant GO terms for biological process in which hub genes were most enriched as well. The most significant GO annotation was cytoplasm for cellular component and protein binding for molecular function, respectively (Supplementary Figures 2B–D).

### Selection of Biomarkers With Prognostic Value

**Figure 5:**
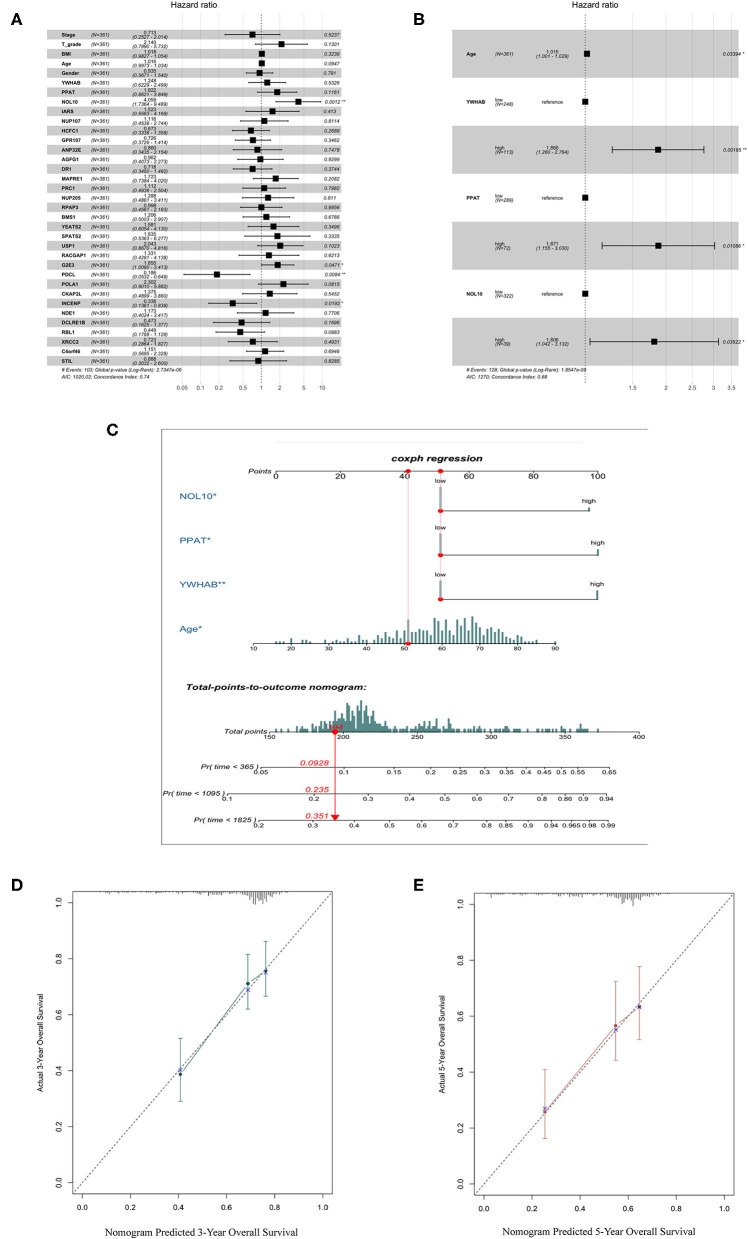
Selection of biomarkers with diagnostic and prognostic value. **(A)** Forest plot of first step selection based on the multivariate Cox regression model. **(B)** Forest plot of final model constructed by forward stepwise strategy. **(C)** Nomogram to predict survival probability at 1, 3, 5 years. **(D)** Calibration curve for the nomogram predicting 3-year overall survival. **(E)** Calibration curve for the nomogram predicting 5-year overall survival.

We selected thirty genes that had never been proved to be associated with HCC from forty hub genes as candidates to construct biomarkers with prognostic value. The dichotomous variables of candidate gene expression and clinicopathological characteristics, such as tumor stage, T grade, body mass index (BMI), age, and gender, were included as covariates in the multivariate Cox regression model. The result revealed NOL10 expression was the most significant independent factor (Figure 5A). Subsequently, forward stepwise selection was performed and each step chose the most significant gene into the final model. Detailed results of selection at each step was shown in Supplementary Table 1. YWHAB, PPAT, NOL10, and age were eventually considered as independent prognostic factors (Figure 5B). We further combined four independent variables to build a nomogram that visualized their prognostic value in predicting overall survival at 1, 3, and 5 years (Figure 5C). Moreover, calibration curves indicated that the nomogram had good prediction accuracy for 3 and 5-year overall survival (Figures 5D,E). After comprehensive assessment of prognostic value, YWHAB, PPAT, and NOL10 were considered as prognostic biomarkers for HCC.

### Validation of Correlations Between Biomarker Expression and Progression of HCC

**Figure 6:**
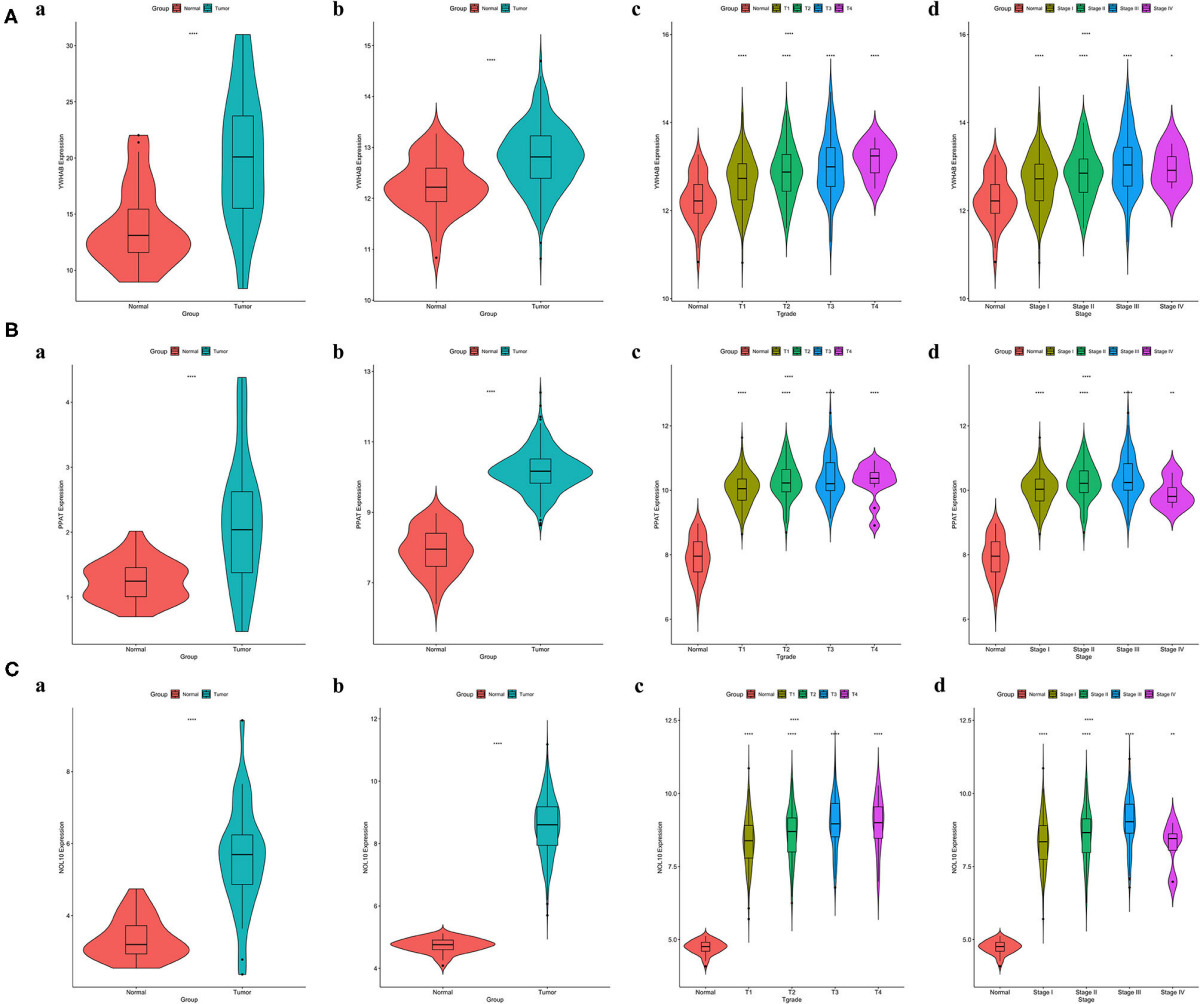
Correlations between biomarker expression and progression of HCC. **(A)** YWHAB expression differences. **(B)** PPAT expression differences. **(C)** NOL10 expression differences. (a) between tumor and adjacent normal tissues in the FAHMSZU cohort. (b) between tumor and solid normal tissues in the TCGA-LIHC dataset. (c) between different T grades tumor and solid normal tissues in the TCGA-LIHC dataset. (d) between different stages tumor and solid normal tissues in the TCGA-LIHC dataset. (ns *P* ≥ 0.05; ^*^P < 0.05; ^**^P < 0.01; ^****^P < 0.0001).

It was worth noting that biomarker expression was significantly upregulated in tumor samples compared to adjacent normal controls (*P* < 0.001). The similar results were obtained from a cohort of 32 paired HCC patients treated in our hospital, The First Affiliated Hospital of Medical School of Zhejiang University (FAHMSZU). In comparisons with normal tissue, significant differences in biomarker expression at different T grades and stages were observed. As the tumor stage progressed from early to advanced or the tumor grade progressed from low to high, biomarker expression tended to rise (Figure 6).

### Association of Biomarker Expression With Methylation, Immune Infiltration, and Tumor Proliferation

**Figure 7:**
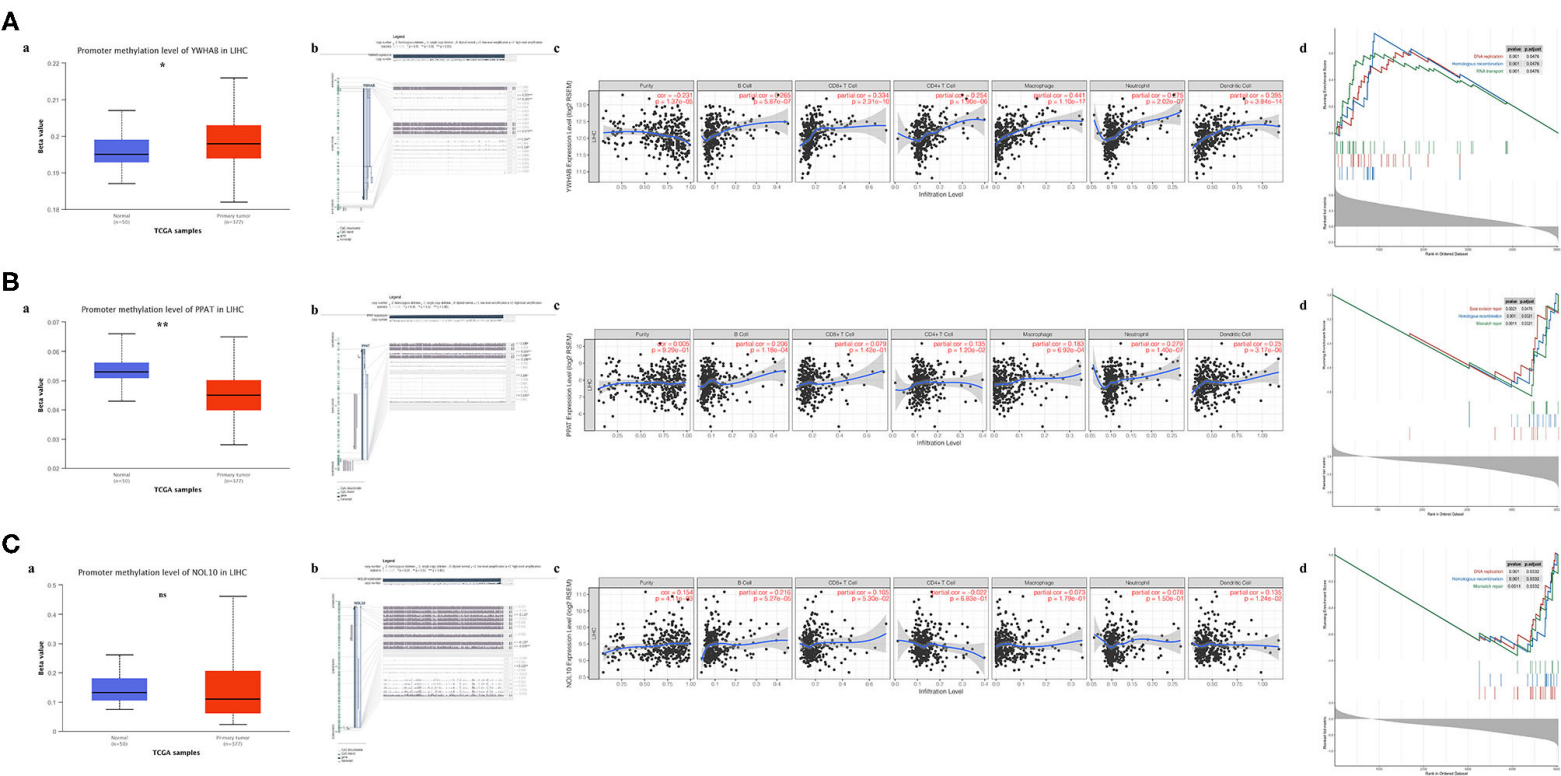
Association of biomarker expression with methylation, immune infiltration and tumor proliferation in the TCGA-LIHC dataset. **(A)** YWHAB, **(B)** PPAT, and **(C)** NOL10. (a) Differences in promoter methylation levels between tumor and solid normal tissues. (b) Association between expression and methylation sites of DNA sequences. (c) Association between expression and immune infiltration. (d) Top 3 enriched KEGG pathways in the high-expression group. (ns *P* ≥ 0.05; ^*^P < 0.05; ^**^P < 0.01).

Regulation of promoter methylation varied when comparing average methylation levels of biomarker in tumors and precancerous normal tissues. Among three biomarkers, YWHAB methylation level was significantly higher in HCC than in normal and PPAT methylation level showed an inverse significant regulation compared to YWHAB while NOL10 methylation level did not differ significantly between two groups. Despite the aforementioned differences in methylation regulation, the similarity was that the expression levels of YWHAB, PPAT, and NOL10 correlated with methylation levels at many methylation sites in their DNA sequences. Regarding immune infiltration, YWHAB, PPAT, and NOL10 were all positively related to B cells and dendritic cells. Notably, positive associations were observed between YWHAB and all of immune infiltration as well as tumor purity. In addition, the results of GSEA plots revealed “DNA replication,” “Homologous recombination” and “Mismatch repair” were three pathways in which genes were significantly enriched. These enriched pathways were all closely related to tumor proliferation (Figure 7).

### Biomarkers Regulate HCC Cell Proliferation and Migration

**Figure 8:**
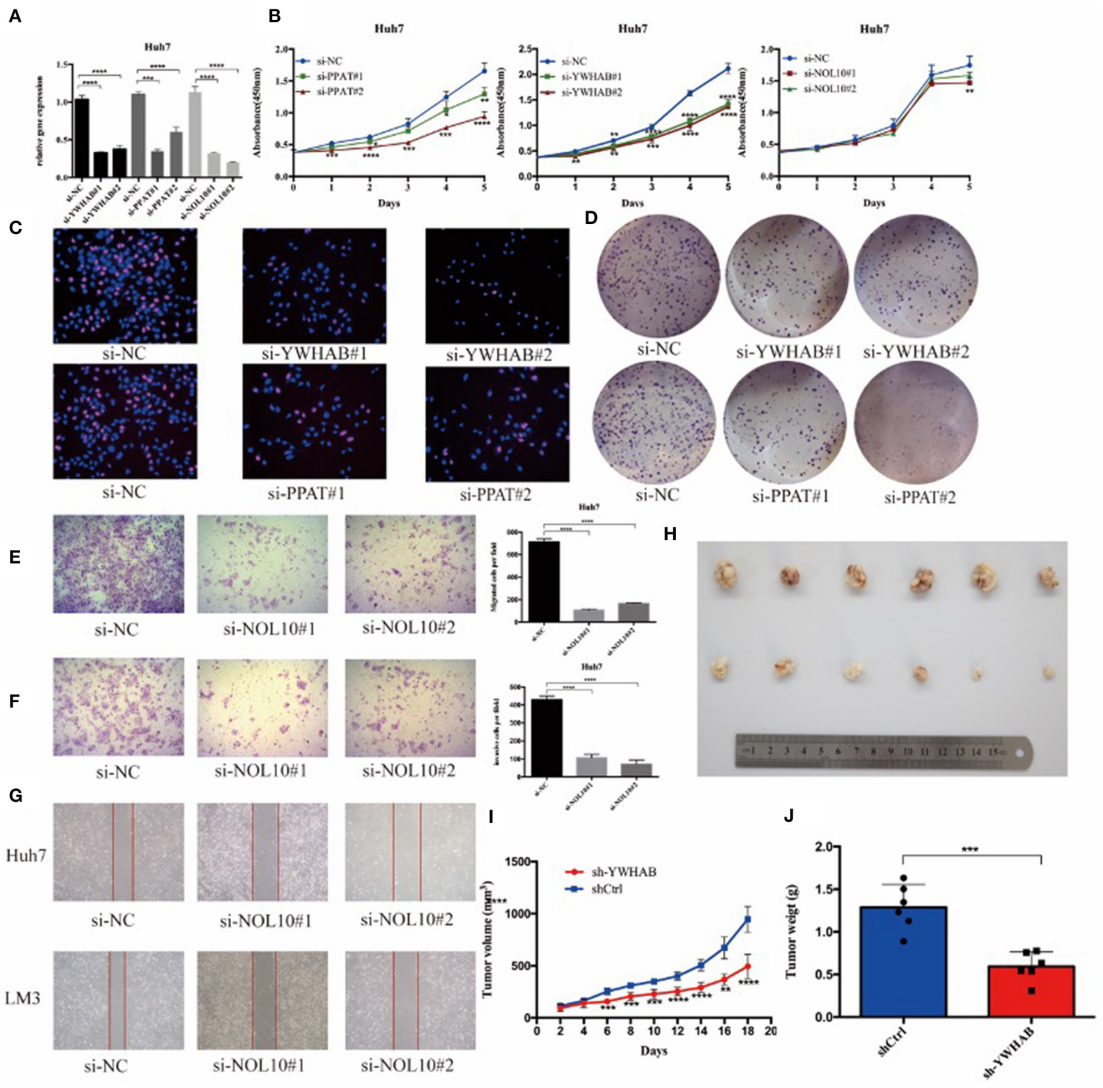
Biomarkers regulate HCC cell proliferation and migration *in vitro* and *in vivo*. **(A)** HCC Huh7 was transfected with four siRNAs. qRT-PCR was used to detect the transfection efficiency. **(B)** CCK-8 assays were conducted to examine Huh7 cell viability after the knockdown of YWHAB, PPAT, and NOL10 expression. **(C)** EdU incorporation assays were used to examine cell proliferation (red signal). The cell nuclei were counterstained with Hoechst (blue signal). Representative images and quantification are shown. **(D)** Knocking down YWHAB and PPAT expression inhibited colony formation in HCC cells. **(E,F)** Migration and invasion of Huh7 was detected after transfection with si-NOL10, respectively, for 24 and 48 h. **(G)** Migration of Huh7 cells was detected after transfection with si-NOL10 for 24. **(H)** Photographs of the subcutaneous tumors are shown. **(I,J)** The tumor weights and volumes were measured. (ns *P* ≥ 0.05; ^*^P < 0.05; ^**^P < 0.01; ^***^P < 0.001; ^****^P < 0.0001).

In order to verify the results of above bioinformatic analysis, we next examined the influence of three biomarkers on the biological behaviors of HCC cell lines. According to the expression levels (Supplementary Figures 4A–C), the HCC-LM3 and Huh7 cell lines were chosen for stable knockdown experiments. YWHAB, PPAT, NOL10 were knocked down in Huh7 and LM3 HCC cell lines, respectively. As shown in Figure 8A, Supplementary Figures 3A, 4D the transfection efficiency was examined by RT-PCR and western blot, CCK-8 assays indicated that knocking down PPAT, YWHAB expression suppressed the proliferative ability of HCC cells, while the effect of NOL10 on HCC growth is insignificant (Figure 8B and Supplementary Figure 3B). In addition, a similar result was also observed with an EdU assay (Figure 8C and Supplementary Figure 3C). Furthermore, it turned out that colony formation in HCC cells was significantly reduced after PPAT and YWHAB depletion (Figure 8D). Next, we found that Huh7 and LM3 cell migration and invasion were observably impaired after NOL10 knockdown (Figures 8E–G). To determine the effect of hub genes on HCC growth *in vivo*, we subcutaneously injected YWHAB-silenced or control LM3 cells into nude mice. All tumors were collected 3 weeks after injection (Figure 8H). The tumor volumes and weights were quantified. The data indicated that silencing YWHAB reduced tumor weight and tumor volume (Figures 8I,J).

### Validation of Diagnostic and Prognostic Value of Biomarkers

**Figure 9:**
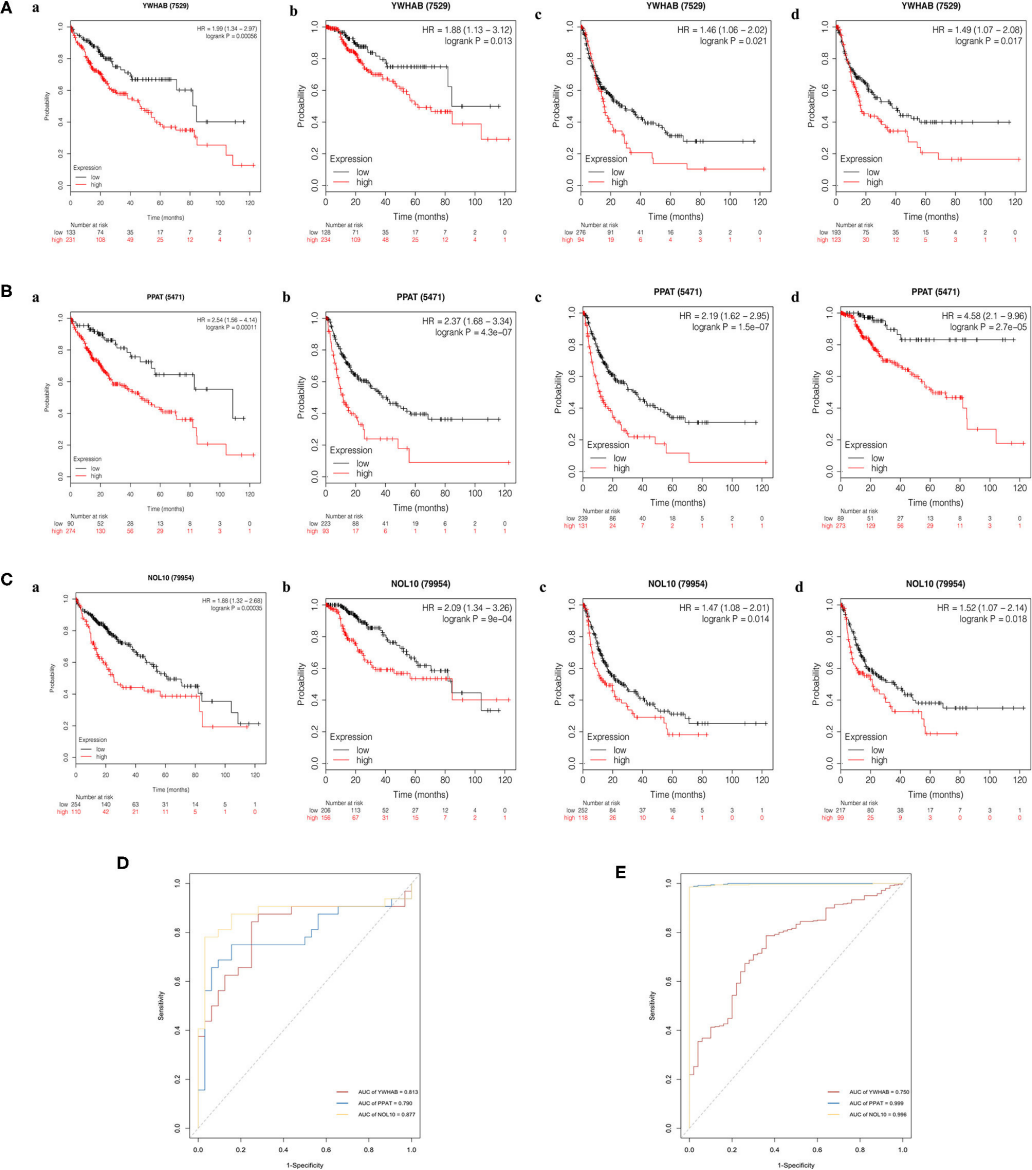
Kaplan–Meier survival analysis of high- and low- expression samples. **(A)** YWHAB, **(B)** PPAT, and **(C)** NOL10. (a) Overall survival. (b) Disease-specific survival. (c) Post-progression survival. (d) Relapse-free survival. **(D)** Diagnostic ROC curves for YWHAB, PPAT, and NOL10 in the FAHMSZU cohort. **(E)** Diagnostic ROC curves for YWHAB, PPAT, and NOL10 in the TCGA-LIHC dataset.

The Kaplan–Meier (KM) plotter was used to validate the prognostic value of biomarker expression. We found that higher expression of three biomarkers was significantly associated with poorer overall survival, relapse-free survival, post-progression survival, and disease-specific survival, suggesting that YWHAB, PPAT, and NOL10 were reliable biomarkers for HCC prognosis (Figures 9A–C). Moreover, high diagnostic value of these three biomarkers was confirmed in the ROC curves depicted in the TCGA-LIHC dataset and the FAHMSZU cohort (Figures 9D,E).

## Discussion

Globally, liver cancer is the fourth leading cause of cancer-related deaths, with the sixth highest incidence (1). HCC accounts for about 80% of all cases of primary liver cancer, most of which occur in patients with chronic diseases such as hepatitis B virus (HBV), hepatitis C virus (HCV) and alcoholism (25, 26). The increase in deaths caused by HCC is a growing concern. Despite significant advances in the early treatment of HCC patients through surgery, the five-year overall survival rate is only 50–70% (5). According to the World Health Organization report, more than one million patients will die from liver cancer by 2030. Thanks to comprehensive genomic analysis and advances in bioinformatics technology, more molecular events have been discovered to study new biomarkers and therapeutic targets for HCC. For instance, many abnormally regulated signaling pathways (such as RAS–MAPK, mTOR, and WNT signaling) and the most frequently mutated drivers (such as TERT promoter, CTNNB1 and TP53) have been confirmed to be associated with HCC (11, 27, 28). Nevertheless, the research achievements of molecular pathogenesis have not yet transformed into molecular therapies for precision medicine (29, 30). Therefore, there is an urgent need to find more effective diagnostic and prognostic markers.

In our study, we adopted three methods of RRA, WGCNA, and multivariate Cox regression analysis to explore novel biomarkers. First, a total of 389 integrated DEGs were identified between HCC samples and normal in 10 GEO datasets based on the RRA analysis. The results of enrichment analysis in multiple GO terms and KEGG pathways revealed that these DEGs had close associations with the progression of HCC due to their robust relations to oxidation-reduction process, zinc ion binding, protein binding, and metabolic pathway. Notably, protein binding is a critical factor in the advance of HCC. Metabolic pathway plays an important role in drug metabolism and may be a risk indicator for the common complications of HCC (31). Furthermore, forty hub genes in the key module were identified through gene co-expression network. Some of these genes, such as FUBP1 (32) and TRIM (33), have been reported to be correlated with the poor prognosis of HCC. Additionally, viral process is the most enriched biological progress GO term of all hub genes. However, how genetic alterations drive cancer phenotypes in HBV/HCV-related HCC remains unknown although viral hepatitis (especially chronic HBV and HCV infection) is the most common cause of HCC, accounting for 80% of HCC cases worldwide (34).

To further search for the effective biomarkers with diagnostic and prognostic value, a multivariate Cox regression model including hub gene expression and clinicopathological characteristics was constructed. As a result, three biomarkers were selected, namely YWHAB, PPAT, and NOL10. Phosphoribosyl pyrophosphate aminotransferase (PPAT) is a key regulatory enzyme in *de novo* purine nucleotide biosynthesis and has become an attractive and credible drug target for leukemia and other cancer therapeutics (35, 36), but its function in HCC remains unclear. Previous studies have reported that circNOL10 is involved in cell proliferation and cell cycle progression of lung cancer through the methylation of splicing factor epithelial splicing regulatory protein 1 (ESRP1) (37), but the role of NOL10 in HCC is implicit. Similar to the other two biomarkers, what role YWHAB plays in the progression of HCC remains a mystery while it proves to be a biomarker for idiopathic pulmonary arterial hypertension (IPAH) (38). Significant differences in expression of these three biomarkers between tumor tissues and normal were observed in the TCGA-LIHC dataset and the FAHMSZU cohort, indicating that YWHAB, PPAT, NOL10 were overexpressed, especially in patients with advanced pathological T grade and stage.

Moreover, the HCC microenvironment is a complex ecosystem that fuses a variety of parenchymal cells and immune-related cells, and the success of immune checkpoint suppression in solid tumors highlights the key role of the tumor microenvironment in tumor progression (39, 40). We utilized TIMER to explore the biomarker-related immune infiltration and tumor purity, and close associations with B cells and dendritic cells were detected. One plausible hypothesisis that an altered liver microenvironment, through reprogramming of the inflammatory milieu, may contribute to HCC. Patients with HCC present spontaneous antitumor immunity despite the fact that they arise in a “tolerogenic” environment. Recently, some immune-based approaches such as antigen-based vaccination strategies (peptides, dendritic cells), immune checkpoint blockade, adoptive cell therapy and Toll like receptor (TLR) agonists have been shown in preclinical and clinical development for HCC. Traditional subsets of dendritic cells (DC) (cDC1 and cDC2) have been reported to be inhibited due to local factors (including IL-10), which prevented mature DCs from inducing anti-tumor immune responses in HCC (41, 42). A recent research has shown that upregulated β-catenin (CTNNB1) promotes immune escape and involves defects in recruitment of dendritic cells (43). In our work, the positive correlations between biomarkers and immune cells suggested YWHAB, PPAT, and NOL10 likely related to tumor microenvironment, which affected the recruitment of infiltrating immune cells into the tumor microenvironment of HCC. However, little is known about the characteristics and functions of biomarkers and immune cells (B cells and DC subsets) in patients with HCC. It requires more work to confirm in the future.

To further uncover the potential biological functions of biomarkers in HCC, we performed GSEA analysis on the high expression groups of each biomarker and found that “DNA replication,” “Homologous recombination” and “Mismatch repair” were three significantly enriched pathways. Homologous recombination is the main pathway to repair DNA double-strand breaks, the most fatal form of DNA damage. Based on the findings from GSEA, we proposed that YWHAB, PPAT, and NOL10 made great contributions to the proliferation of HCC. We further knocked down the expression of PPAT, YWHAB, and NOL10 in HCC cells and found that depletion of PPAT, YWHAB suppressed the tumor proliferation. The effect of YWHAB on HCC growth was also observed *in vivo*. Moreover, NOL10 silencing inhibited the migration and invasion of HCC. Interestingly, higher expression of three biomarkers has robust connections with poorer overall survival, relapse-free survival, post-progression survival, and disease-specific survival, indicating that YWHAB, PPAT, and NOL10 are reliable biomarkers to predict prognosis in patients with HCC. Based on the ROC curves, YWHAB, PPAT, and NOL10 have also been identified as potential biomarkers for diagnosing HCC with high sensitivity and specificity. The above findings from bioinformatics analysis and laboratory experiments demonstrated that novel biomarkers we selected performed well for survival prediction and diagnosis of HCC.

## Data Availability Statement

Publicly available datasets were analyzed in this study, these can be found in The Cancer Genome Atlas (https://portal.gdc.cancer.gov/); the NCBI Gene Expression Omnibus (GSE14520, GSE22405, GSE62232, GSE59261, GSE101685, GSE121248, GSE64041, GSE50579, and GSE25097). The datasets generated in this study can be found in the NCBI Gene Expression Omnibus (GSE138485).

## Ethics Statement

The studies involving human participants and vertebrate animals were reviewed and approved by the Ethical Review Committee of First Affiliated Hospital, School of Medicine, Zhejiang University. The patients/participants and legal guardian/next of kin provided written informed consent to participate in this study.

## Author Contributions

XH, MB, LZ, and SZ: conceptualization. MB and JH: data curation and software. XH and MB: formal analysis, visualization, investigation, and writing—original draft. LZ and SZ: funding acquisition and project administration. XH, MB, and JH: methodology. XH: resources and validation. MB: supervision. XH, LZ, and SZ: writing—review and editing. All authors: contributed to the article and approved the submitted version.

## Conflict of Interest

The authors declare that the research was conducted in the absence of any commercial or financial relationships that could be construed as a potential conflict of interest.
