# Corrigendum: Identification and Validation of Novel Biomarkers for Diagnosis and Prognosis of Hepatocellular Carcinoma

**DOI:** 10.3389/fonc.2020.617539

**Published:** 2020-11-20

**Authors:** Xiaoyi Hu, Mingyang Bao, Jiacheng Huang, Lin Zhou, Shusen Zheng

**Affiliations:** ^1^ Division of Hepatobiliary and Pancreatic Surgery, Department of Surgery, The First Affiliated Hospital, Zhejiang University School of Medicine, Hangzhou, China; ^2^ National Health and Family Planning Commission of China Key Laboratory of Combined Multi-Organ Transplantation, The First Affiliated Hospital, Zhejiang University School of Medicine, Hangzhou, China; ^3^ Key Laboratory of the Diagnosis and Treatment of Organ Transplantation, Research Unit of Collaborative Diagnosis and Treatment for Hepatobiliary and Pancreatic Cancer, Chinese Academy of Medical Sciences, Hangzhou, China; ^4^ Key Laboratory of Organ Transplantation, The First Affiliated Hospital, Zhejiang University School of Medicine, Hangzhou, China; ^5^ State Key Laboratory of Genetic Engineering, School of Life Sciences, Institute of Biostatistics, Fudan University, Shanghai, China

**Keywords:** prognosis, biomarker, GEO, hepatocellular carcinoma (HCC), diagnosis

In the published article, there was an error in affiliation 2, “State Key Laboratory of Genetic Engineering, School of Life Sciences, Institute of Biostatistics, Fudan University, Shanghai, China.” Instead of affiliation 2, it should be affiliation 5.

The authors apologize for this error and state that this does not change the scientific conclusions of the article in any way. The original article has been updated.

