# Supplementary material for: Identification and Validation of Novel Biomarkers for Diagnosis and Prognosis of Hepatocellular Carcinoma
Source: Front Oncol. 2020 Sep 25;10:541479. doi: 10.3389/fonc.2020.541479 (PMC7545743; doi:10.3389/fonc.2020.541479)
Supplement: Supplementary file 1 [file Data_Sheet_1.docx]

Supplementary file


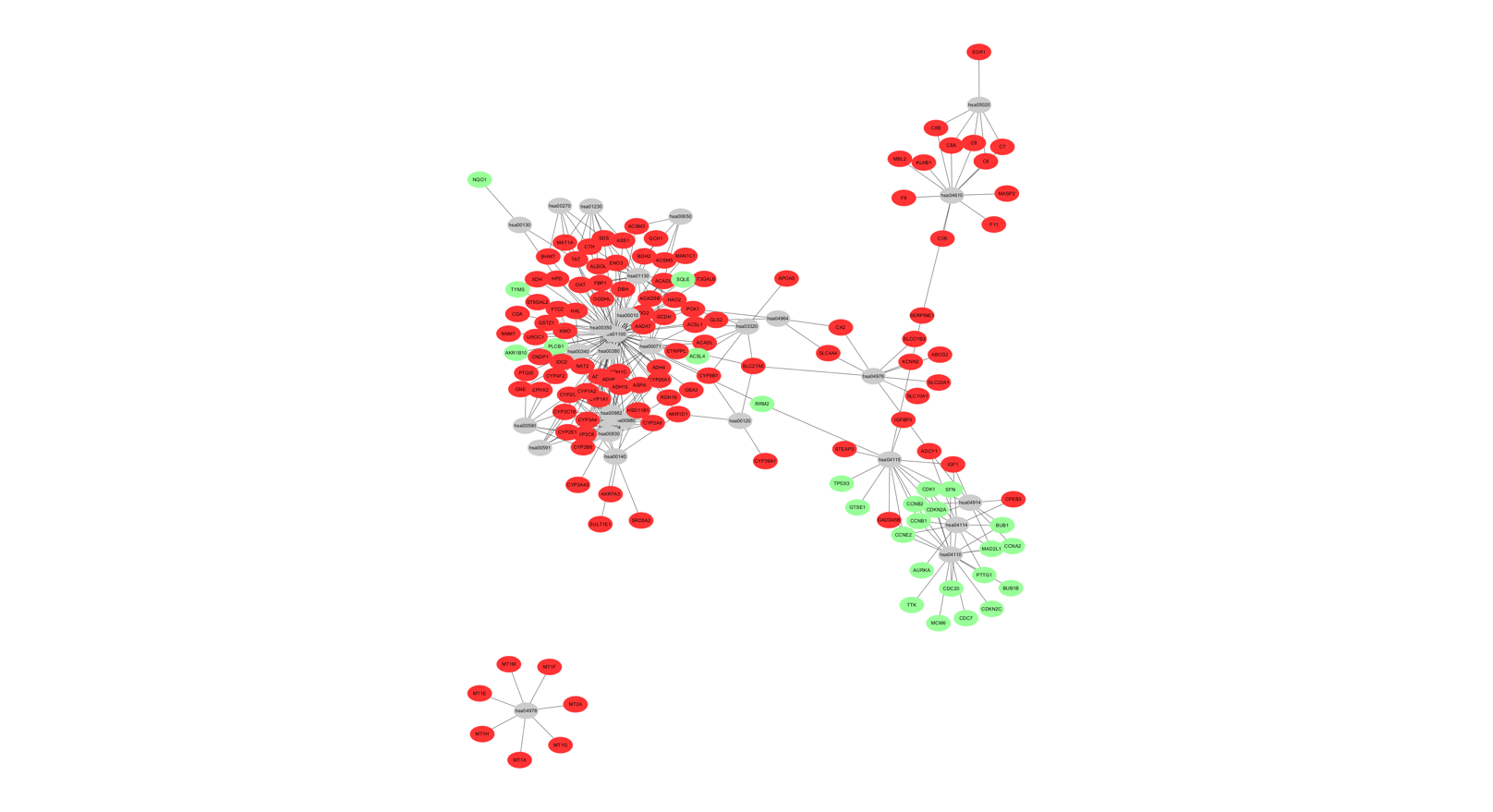


Figure S1 Network map of enriched KEGG pathways. Gray ellipses represent the KEGG pathways, red ellipses represent the upregulated DEGs and green ellipses represent the downregulated DEGs.


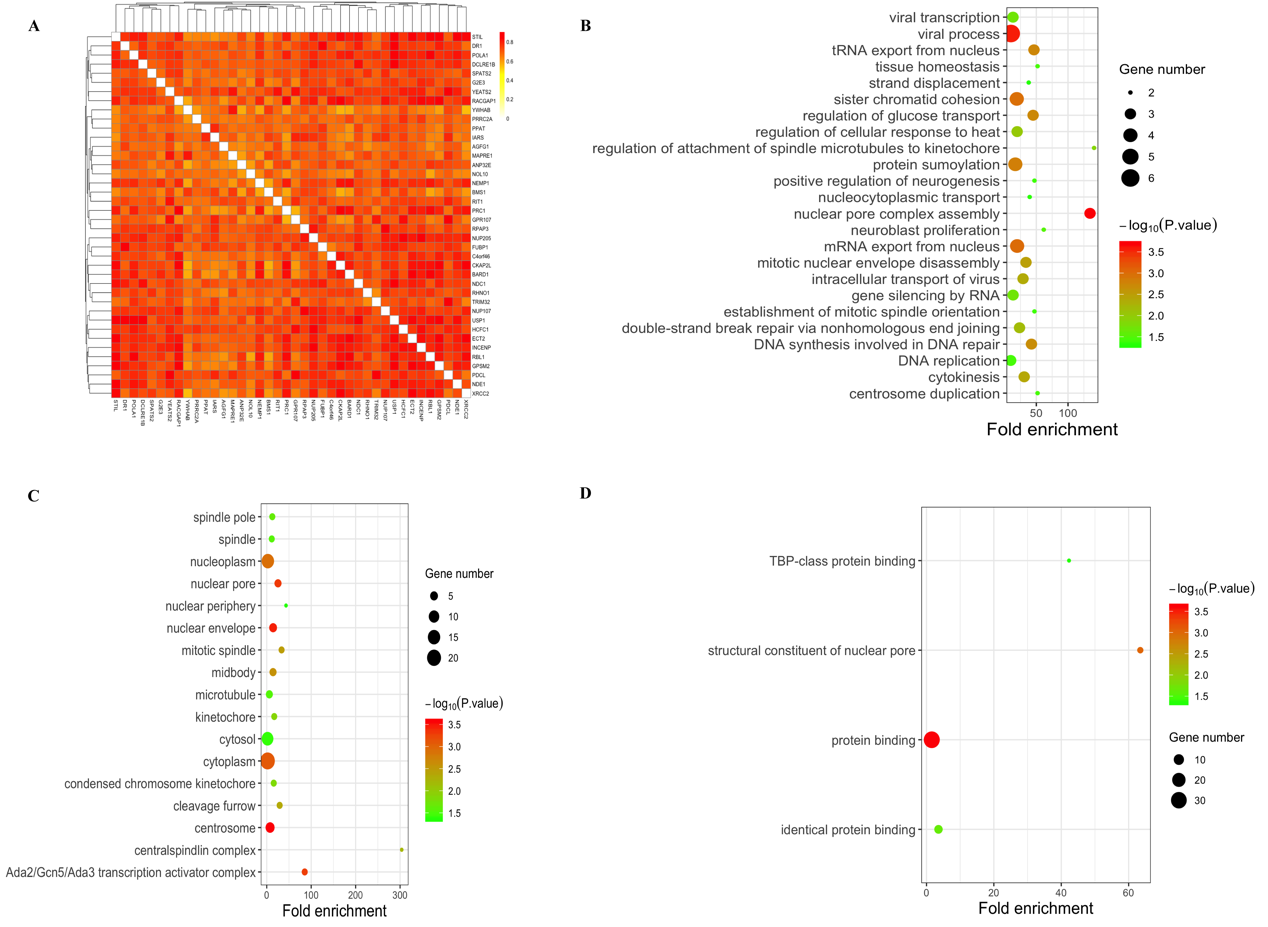


Figure S2 Functional annotation of hub genes. (A) Close correlation between hub genes. (B) Biological process GO terms for hub genes. (C) Cellular component GO terms for hub genes. (D) Molecular function GO terms for hub genes.

Table S1 Top 30 enriched GO terms associated with upregulated genes

| Category | Term | Count | P-value |
| --- | --- | --- | --- |
| Biological Process | GO:0055114~oxidation-reduction process | 39 | 2.28E-13 |
| Biological Process | GO:0006508~proteolysis | 20 | 4.46E-04 |
| Biological Process | GO:0042493~response to drug | 17 | 3.19E-05 |
| Biological Process | GO:0006955~immune response | 15 | 0.007853 |
| Biological Process | GO:0006805~xenobiotic metabolic process | 13 | 3.64E-09 |
| Biological Process | GO:0006954~inflammatory response | 13 | 0.018768 |
| Biological Process | GO:0019373~epoxygenase P450 pathway | 12 | 3.98E-16 |
| Biological Process | GO:0017144~drug metabolic process | 12 | 1.45E-13 |
| Biological Process | GO:0006956~complement activation | 12 | 1.34E-07 |
| Biological Process | GO:0007166~cell surface receptor signaling pathway | 11 | 0.012852 |
| Cellular Components | GO:0070062~extracellular exosome | 87 | 3.74E-11 |
| Cellular Components | GO:0005576~extracellular region | 66 | 1.57E-13 |
| Cellular Components | GO:0005615~extracellular space | 49 | 2.48E-08 |
| Cellular Components | GO:0005887~integral component of plasma membrane | 39 | 4.28E-04 |
| Cellular Components | GO:0005789~endoplasmic reticulum membrane | 28 | 3.13E-04 |
| Cellular Components | GO:0031090~organelle membrane | 17 | 2.37E-13 |
| Cellular Components | GO:0005759~mitochondrial matrix | 14 | 0.001566 |
| Cellular Components | GO:0072562~blood microparticle | 13 | 3.69E-06 |
| Cellular Components | GO:0016323~basolateral plasma membrane | 10 | 0.001773 |
| Cellular Components | GO:0005581~collagen trimer | 7 | 0.002796 |
| Molecular Function | GO:0008270~zinc ion binding | 30 | 0.008376 |
| Molecular Function | GO:0020037~heme binding | 21 | 3.27E-14 |
| Molecular Function | GO:0005506~iron ion binding | 21 | 2.80E-13 |
| Molecular Function | GO:0042803~protein homodimerization activity | 20 | 0.019379 |
| Molecular Function | GO:0016705~oxidoreductase activity, acting on paired donors, with incorporation or reduction of molecular oxygen | 16 | 6.02E-15 |
| Molecular Function | GO:0004497~monooxygenase activity | 16 | 8.03E-15 |
| Molecular Function | GO:0016491~oxidoreductase activity | 15 | 3.02E-06 |
| Molecular Function | GO:0004252~serine-type endopeptidase activity | 15 | 4.70E-05 |
| Molecular Function | GO:0019825~oxygen binding | 14 | 1.98E-13 |
| Molecular Function | GO:0003824~catalytic activity | 14 | 7.78E-06 |

Table S2 Top 30 enriched GO terms associated with downregulated genes

| Category | Term | Count | P-value |
| --- | --- | --- | --- |
| Biological Process | GO:0051301~cell division | 24 | 2.14E-18 |
| Biological Process | GO:0007067~mitotic nuclear division | 19 | 3.01E-15 |
| Biological Process | GO:0008283~cell proliferation | 13 | 1.16E-06 |
| Biological Process | GO:0007062~sister chromatid cohesion | 11 | 3.52E-10 |
| Biological Process | GO:0000086~G2/M transition of mitotic cell cycle | 11 | 5.83E-09 |
| Biological Process | GO:0000082~G1/S transition of mitotic cell cycle | 10 | 6.58E-09 |
| Biological Process | GO:0006915~apoptotic process | 10 | 0.005126 |
| Biological Process | GO:0042787~protein ubiquitination involved in ubiquitin-dependent protein catabolic process | 9 | 2.66E-06 |
| Biological Process | GO:0031145~anaphase-promoting complex-dependent catabolic process | 8 | 3.31E-07 |
| Biological Process | GO:0051726~regulation of cell cycle | 8 | 6.99E-06 |
| Cellular Components | GO:0005634~nucleus | 56 | 1.66E-08 |
| Cellular Components | GO:0005737~cytoplasm | 54 | 4.33E-08 |
| Cellular Components | GO:0005829~cytosol | 46 | 1.08E-10 |
| Cellular Components | GO:0005654~nucleoplasm | 37 | 8.26E-08 |
| Cellular Components | GO:0016020~membrane | 21 | 0.009893 |
| Cellular Components | GO:0030496~midbody | 12 | 7.69E-11 |
| Cellular Components | GO:0005874~microtubule | 11 | 5.59E-06 |
| Cellular Components | GO:0000777~condensed chromosome kinetochore | 10 | 7.79E-10 |
| Cellular Components | GO:0005813~centrosome | 10 | 4.12E-04 |
| Cellular Components | GO:0048471~perinuclear region of cytoplasm | 10 | 0.005447 |
| Molecular Function | GO:0005515~protein binding | 76 | 2.08E-08 |
| Molecular Function | GO:0005524~ATP binding | 23 | 1.83E-05 |
| Molecular Function | GO:0019901~protein kinase binding | 14 | 1.59E-07 |
| Molecular Function | GO:0042803~protein homodimerization activity | 12 | 0.002416 |
| Molecular Function | GO:0042802~identical protein binding | 11 | 0.008750 |
| Molecular Function | GO:0004674~protein serine/threonine kinase activity | 9 | 0.001182 |
| Molecular Function | GO:0008017~microtubule binding | 8 | 1.61E-04 |
| Molecular Function | GO:0004672~protein kinase activity | 8 | 0.003901 |
| Molecular Function | GO:0003682~chromatin binding | 7 | 0.021980 |
| Molecular Function | GO:0016301~kinase activity | 5 | 0.045661 |

Table S3 Significant enriched KEGG pathways

| Pathway | ID | Count | P-value |
| --- | --- | --- | --- |
| Metabolic pathways | hsa01100 | 74 | 1.08E-09 |
| Chemical carcinogenesis | hsa05204 | 16 | 1.22E-08 |
| Retinol metabolism | hsa00830 | 14 | 4.34E-08 |
| p53 signaling pathway | hsa04115 | 14 | 7.73E-08 |
| Metabolism of xenobiotics by cytochrome P450 | hsa00980 | 14 | 2.64E-07 |
| Fatty acid degradation | hsa00071 | 11 | 3.40E-07 |
| Drug metabolism - cytochrome P450 | hsa00982 | 13 | 7.35E-07 |
| Cell cycle | hsa04110 | 17 | 8.88E-07 |
| Complement and coagulation cascades | hsa04610 | 12 | 6.08E-06 |
| Tyrosine metabolism | hsa00350 | 9 | 7.37E-06 |
| Tryptophan metabolism | hsa00380 | 8 | 1.72E-04 |
| Oocyte meiosis | hsa04114 | 12 | 5.16E-04 |
| Glycolysis / Gluconeogenesis | hsa00010 | 9 | 8.87E-04 |
| Bile secretion | hsa04976 | 9 | 0.001079 |
| Linoleic acid metabolism | hsa00591 | 6 | 0.001616 |
| Steroid hormone biosynthesis | hsa00140 | 8 | 0.001743 |
| Mineral absorption | hsa04978 | 7 | 0.00197 |
| Arachidonic acid metabolism | hsa00590 | 8 | 0.002342 |
| Prion diseases | hsa05020 | 6 | 0.003347 |
| Histidine metabolism | hsa00340 | 5 | 0.003938 |
| Progesterone-mediated oocyte maturation | hsa04914 | 9 | 0.004737 |
| Biosynthesis of antibiotics | hsa01130 | 14 | 0.012239 |
| Primary bile acid biosynthesis | hsa00120 | 4 | 0.013723 |
| PPAR signaling pathway | hsa03320 | 7 | 0.015662 |
| Biosynthesis of amino acids | hsa01230 | 7 | 0.021661 |
| Cysteine and methionine metabolism | hsa00270 | 5 | 0.02727 |
| Proximal tubule bicarbonate reclamation | hsa04964 | 4 | 0.03129 |
| Ubiquinone and other terpenoid-quinone biosynthesis | hsa00130 | 3 | 0.042184 |
| Butanoate metabolism | hsa00650 | 4 | 0.047329 |

Table S4 Multivariate Cox regression with forward stepwise selection

| Step | Selected variable | B | SE | Wald | P-value | HR (95%CI) |
| --- | --- | --- | --- | --- | --- | --- |
| 1 | NOL10 | 1.465 | 0.246 | 35.359 | 0.001 | 4.059 (1.736~9.489) |
| 2 | YWHAB | 0.690 | 0.214 | 10.400 | 0.002 | 1.993 (1.311~3.031) |
|  | NOL10 | 1.092 | 0.270 | 16.308 | 0.001 | 2.979 (1.754~5.060) |
| 3 | YWHAB | 0.579 | 0.223 | 6.744 | 0.009 | 1.784 (1.153~2.762) |
|  | NOL10 | 0.864 | 0.291 | 8.841 | 0.003 | 2.372 (1.342~4.193) |
|  | PPAT | 0.590 | 0.251 | 5.510 | 0.019 | 1.804 (1.102~2.952) |
| 4 | Age | 0.017 | 0.008 | 3.741 | 0.034 | 1.015 (1.001~1.029) |
|  | YWHAB | 0.900 | 0.222 | 9.322 | 0.002 | 1.866 (1.260~2.764) |
|  | NOL10 | 0.016 | 0.008 | 3.027 | 0.035 | 1.806 (1.042~3.132) |
|  | PPAT | 0.680 | 0.257 | 6.014 | 0.011 | 1.871 (1.155~3.030) |


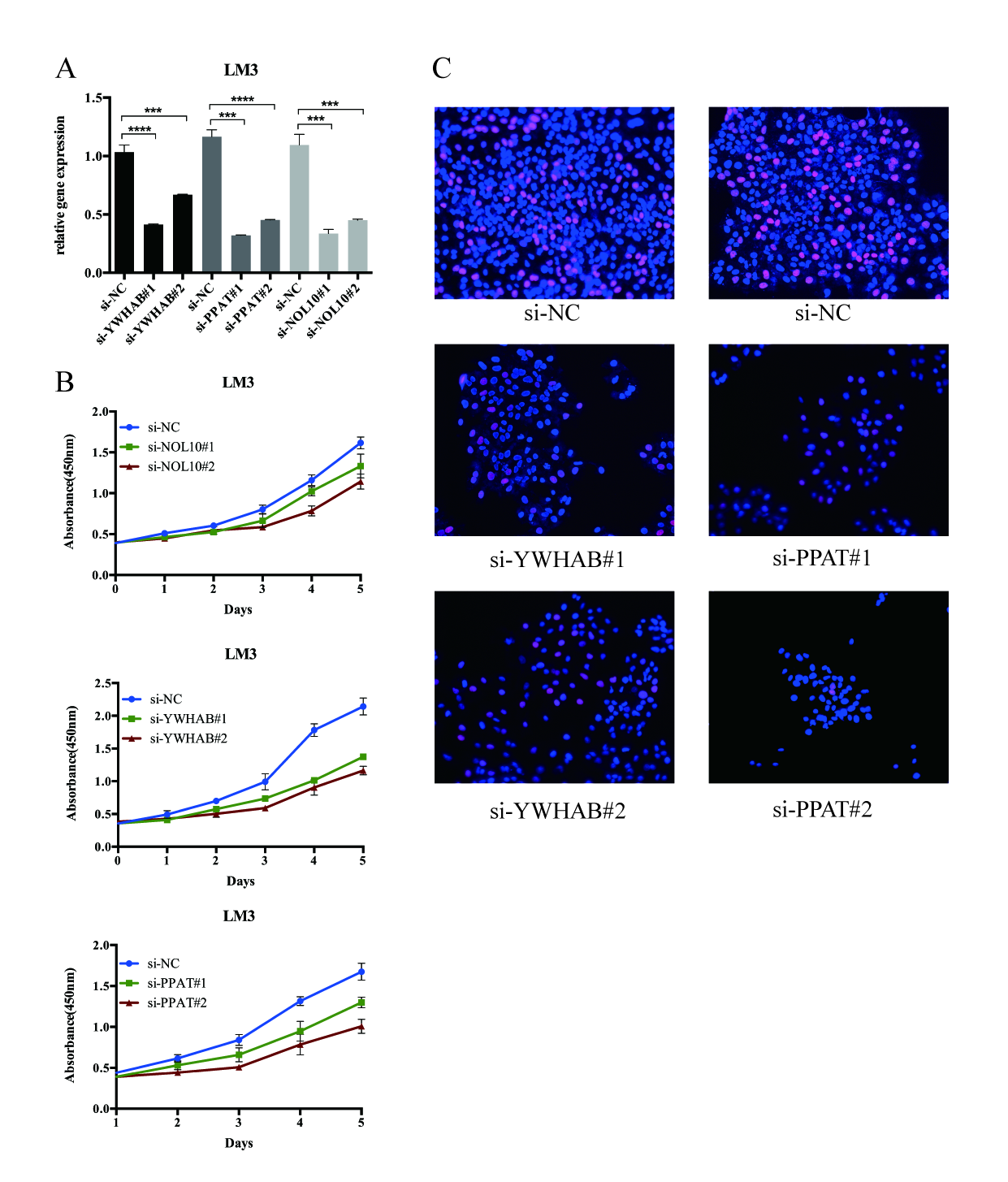


Figure S3 Biomarkers regulate HCC LM3 cell proliferation in vitro. (A) HCC LM3 was transfected with four siRNAs. qRT-PCR was used to detect the transfection efficiency. (B) CCK-8 assays were conducted to examine LM3 cell viability after the knockdown of YWHAB, PPAT and NOL10 expression. (C)EdU incorporation assays were used to examine cell proliferation (red signal). The cell nuclei were counterstained with Hoechst (blue signal). Representative images and quantification are shown.


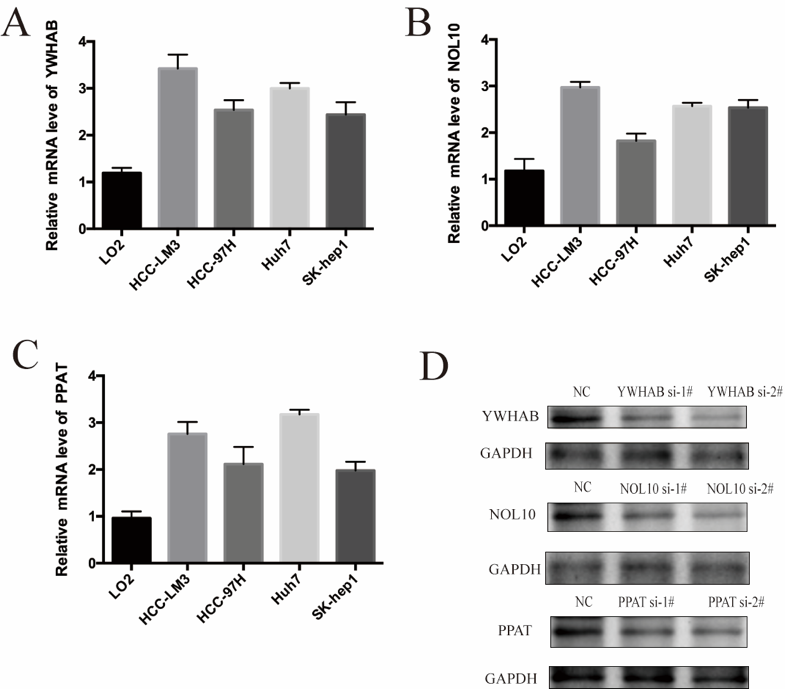


Figure S4 (A-C) mRNA level of YWHAB, NOL10 and PPAT in an immortalized hepatic cell line and four HCC cell lines (D) The efficiency of YWHAB, NOL10 and PPAT knockdown in HCC-LM3 cells
